# Risks for infection of strawberry plants with an aerosolized inoculum of *Xanthomonas fragariae*

**DOI:** 10.1007/s10658-018-1513-9

**Published:** 2018-05-31

**Authors:** J. M. van der Wolf, A. Evenhuis, P. Kastelein, M. C. Krijger, V. Z. Funke, W. van den Berg, A. F. Moene

**Affiliations:** Wageningen University and Research, PO Box 16, 6700 Wageningen, AA Netherlands

**Keywords:** Angular leaf spot, Air sampling, Particle counters, Infection thresholds, TaqMan assay, *Fragaria x ananasa*

## Abstract

*Xanthomonas fragariae* is the causative agent of angular leaf spot of strawberry, a quarantine organism in plant propagation material in the European Union. Field experiments were conducted to assess the risks for infection of strawberry plants through dispersal of an aerosolized inoculum. In practice, pathogen aerosols can be formed during mowing of an infected crop or by water splashing on symptomatic plants during overhead irrigation or rain. In our experiments, aerosols were generated by spraying suspensions of *X. fragariae* with a density of 10^8^ cfu ml^−1^ or water under pressure vertically up into the air. In strawberry plants (cv Elsanta) placed at 1.3, 5 and 10 m distance downwind from the spray boom, infections were found, as evidenced with a combination of dilution–plating and molecular techniques, but more frequently in plants wetted prior to inoculation than in plants kept dry. A logarithmic decrease in infection incidence was found with the distance to the inoculum source. Symptomatic plants were found up to 5 m distance from the inoculum source. No infected plants were found in plants placed 4 m upwind or treated with water. In glasshouse studies, it was shown that under conditions favorable for disease development, spray-inoculation of strawberry plants with estimated densities of *X. fragariae* as low as 2000 cfu per plant were able to cause symptoms both in cv Elsanta and cv Sonata. Results indicate that there is a considerable risk on infections of strawberry plants exposed to aerosolized inoculum.

## Introduction

*Xanthomonas fragariae* (Kennedy and King 1962a; Kennedy and King 1962b), is the causative agent of angular leaf spot of strawberry. At present, in Europe the pathogen is listed as a quarantine organism on strawberry plants intended for planting (Anonymous 2006). Infections can result in high economic losses as plants should be removed and destroyed upon disease outbreaks (Desmet et al. 2006). In the Netherlands, one of the biggest producers of strawberry planting material in Europe (Lieten 2014), legislation is in place about the radius of the buffer zone to be cleared around disease foci.

Management of the disease is mainly based on exclusion of the pathogen via cultivation practices and hygienic measures. No chemical or biological control agents are currently available to control the disease, but on a small scale, thermotherapy is applied to reduce infection pressure (Turechek and Peres 2009; Van Kruistum et al. 2015). All commercial cultivars are susceptible to *X. fragariae* although at a various level (Desmet et al. 2009; Turechek and Peres 2009).

Strawberry cultivation starts with in vitro material and the first generations of strawberry plants for planting, representing the highest classes, are grown in aphid-free glasshouses, where the risk for infections with *X. fragariae* is considered low (Van der Gaag et al. 2013). In Europe, the last two generations of plant material are often grown in the field which increases the risk for infection.

A field-grown crop that is initially free of the pathogen can become infected via different pathways which may include contact with contaminated machineries, materials, animals, shoes and clothes (Maas 2004), drenching of planting material in preventive fungicide baths (Melis et al. 2012), use of contaminated irrigation water, carry-over contamination from infected crops grown nearby via splashing water, or via aerosols (EPPO 1997; Van der Wolf et al. 2017).

Contaminated aerosols can be generated during splashing of water on symptomatic plants, which can exude large quantities of *X. fragariae* (up to 10^12^ cfu) during spraying of plants with an excess of water (unpublished data). Aerosols can also be generated during mowing of a strawberry crop at the end of the growing season (Van der Wolf et al. 2017). Mowing of strawberry crops is a cultivation practice to lower transpiration rate (Rätsep et al. 2015), to renovate plants after the first harvest (Rätsep et al. 2015) or to remove the excess of leaves prior to low temperature storage of so-called frigo plants.

Dispersal of pathogens via aerosols has been shown to play a role in the epidemiology of various plant pathogenic bacteria including *Pseudomonas syringae* (Morris et al. 2007), *Pectobacterium* and *Dickeya* species (Perombelon et al. 1979; Franc and DeMott 1998) and bacterial pathogens of tomato (McInnes et al. 1988). It was hypothesized that aerosols can be responsible for dissemination over a distance of at least 100 m from the inoculum source (Perombelon et al. 1979). It was found that plant pathogenic bacteria, i.e. Pectobacterium species, can act as cloud condensation nuclei (Franc and DeMott 1998). If aerosolized bacteria are transported into cloud systems, they can move over much larger distances before they will be deposited in precipitation.

Several factors are described that are involved in disease development which include the cultivar (Pérez-Jiménez et al. 2012; Rivera-Zabala et al. 2017), the susceptibility of the plant (Kennedy and King 1962b), the virulence of the pathogen (Rivera-Zabala et al. 2017) and environmental conditions, in particular humidity and temperature (Kennedy and King 1962b; Hildebrand et al. 2005). Long periods of rain, irrigation or dew favour the disease (Maas 1998). The assumption is that leaf wetness is necessary for a successful infection. Moist conditions also favour exudation of the pathogen from lesions (Maas 1998).

The aim of this study was to assess the risks for infections and disease development after spread of aerosolized inoculum onto strawberry plants at various distances from the source. In field experiments bacteria were released and the maximum distance estimated at which dissemination resulted in an infection of strawberry plants. Strawberry plants were either wetted or kept dry prior to inoculation, to vary in leaf wetness conditions. In addition, glasshouse experiments were conducted to determine the minimum inoculum pressure to establish an infection.

## Materials and methods

### *Xanthomonas fragariae* and culturing

A natural Rifampicin resistant strain (designated IPO3488) of *X. fragariae* isolate PD 3145, obtained in 1997 from strawberry in Spain, was used in the field experiments. Strain IPO3488 was kindly supplied by Dr. H. Koenraadt of the Netherlands Inspection Service for Horticulture (Naktuinbouw). In preceding pathogenicity tests, strain 3488 proved as virulent as the parental wild type strain (data not shown).

Strain 3488 was stored on beads (Protect bacterial preservers, TS/70; Technical Service Consultants Ltd., Lancashire, UK) at −80 °C. Three to 4 weeks before starting the experiment the strain was revived on Tryptic Soy Agar (Difco, USA) at 25 °C and maintained at 17 °C by monthly transfers on YDC medium (Duchefa Biochemie, NL) with 50 mg l^−1^ Rifampicin (Duchefa Biochemie). Inoculum was prepared by growing the strain on glycine amended R2A Agar (R2AG: 18.12 g l^−1^ R2A Agar, Difco USA, and 25 mg l^−1^ glycine; Sigma-Aldrich, USA) with 50 mg l^−1^ Rifampicin (Duchefa Biochemie, NL) for 3 days at 25 °C and washing the cells from the agar with a quarter-strength Ringer solution (Oxoid, UK).

To check for the presence of *X*. *fragariae* in air or leaves, 50 μl concentrated air sample or leaf extract was plated undiluted and ten-fold diluted in quarter-strength Ringer solution on R2AGRC; R2AG with 50 mg l^−1^ Rifampicin and 200 mg l^−1^ Cycloheximide (Duchefa Biochemie). Plates were incubated for 8–10 days at 25 °C before inspection for the presence of Xanthomonas-like colonies (circular, convex, glistening and translucent to pale-yellow).

### Glasshouse experiments

Two experiments were conducted to determine the lowest inoculum density needed to cause angular leaf spot in strawberry leaves. The first experiment was conducted in March–April 2013 and the second in October–November 2014. Plants of cultivars Elsanta and Sonata were grown in a glasshouse at 17 °C and 65–70% RH. In 2014, daylight was prolonged to 14 h. Approximately 20 days after planting of the cold (−1.5 °C) stored certified waiting bed plants, 1-L pots containing Lentse potting soil no.3 (Horticoop, NL), two – three fully expanded leaves were present. During plant growth, stolons and inflorescences were dissected from plants. In 2014, plants were treated against mildew with Bupirimate (Nimrod; Adama, IL) according the manufacturer’s instructions. Approximately 35 days after planting, when the first flower branch was dissected, plants were inoculated with different inoculum densities of *X. fragariae*. A stock suspension in 0.3% (*v*/v) Silwet 719 (Momentive, USA) was set to an absorbance value of A600nm = 0.1 (approximately 10^8^ cfu ml^−1^) by diluting bacterial inoculum prepared in the lab to the desired inoculum density. Inoculation was done by atomizing either undiluted, 100 x, 10,000 x, or 1,000,000 x diluted stock suspension onto the abaxial side of the leaves using a high pressure plant sprayer (Gardena, DE). Twenty to 25 ml of bacterial suspension was applied per plant. Mock inoculated plants were sprayed with 0.3% Silwet 719. After inoculation, strawberry plants were placed in a plastic tent for maintaining high moisture conditions. One day after inoculation (dpi), the tent was removed and plants were distributed in the glasshouse in five blocks. At 14 and 28 dpi every leaflet was examined for the presence of symptoms of angular leaf spot. Furthermore, at 28 dpi for each leaflet the percentage of necrotic leaf area was estimated and the severity indexes calculated, i.e. the average percentage necrotic leaf surface times the number of affected leaflets per plant.

Analysis of variance (ANOVA; Genstat 18.1, VSN International, UK) was used to analyse the effect of *X. fragariae* inoculum density on disease incidence and severity in plants for each cultivar separately. For the analysis of the effects on incidences angular-transformed values were used. Duncan’s new multiple range test was used for evaluating the significance of differences between averages within cultivars.

### Field experiment

On 25 May and 13 June 2016, during dry spells on two rainy days, an experiment was conducted to assess the risk for infection of strawberry plants after aerosol dispersal of *X*. f*ragariae*.

### Strawberry plants and cultivation

On two time points in April 2016 cold (−1.5 °C) stored certified waiting bed plants of cv Elsanta were planted in 11x11x12 cm TEKU pots (Pöppelmann, DE) filled with Lentse potting soil. The interval between both planting dates was 2 weeks.

The first 4 weeks after potting the plants were placed in the open air on weed control fabric. Thereafter the plants were grown under a rain shelter with roofing of polyethylene greenhouse film. Initially the plants were kept on benches, but after being used in an aerosol experiment they were placed on the floor of insect-proof nylon cages in the same rain shelter. Till their use in aerosol experiments the plants were watered and fertilized in line with prevailing horticultural standards. After the aerosol experiment the plants in the insect-proof cages were watered only via irrigation mats to avoid splash dispersal of *X. fragariae* by overhead irrigation.

### Experimental site and weather measurement

The experiment were carried out on a well-cut lawn at Nergena experimental farm near Wageningen, the Netherlands in an area of the Netherlands were no strawberries are grown on a commercial scale. A moveable weather station (Decagon Devices Inc., USA; EM50 datalogger) was placed on the experimental site to measure wind speed, wind direction at 50, 111 and 220 cm above the soil level (Davis cup anemometer). Furthermore, air temperature and relative humidity were recorded on site at 150 cm above soil level (Decagon EHT sensor). Data of other atmospheric variables (sunshine duration and global radiation) were obtained from the Veenkampen weather station at 2.9 km beeline distance from the experimental site.

### Release of inoculum

One day in the experiment consisted of successively a dummy run, a run with spraying tap water and two runs with spraying *X. fragariae* suspensions into the air. About 15 to 20 min before the release of aerosols, air was sampled for 10 min to investigate if *X. fragariae* was naturally present in the air. Thereafter, on each day one 2 L portion of tap water and two 2 L portions of *X. fragariae* suspension set to an absorbance value of A600nm = 0.1 (approximately 10^8^ colony-forming units ml^−1^), were sprayed vertically up into the air with a spray boom kept in inverted position. Inoculum was sprayed with a pressure of 3 bar through 6 Teejet XR 110–02 VP extended rate flat spray nozzles (Teejet Technologies, USA) with in-between distances of 60 cm. The spray boom was situated at a height of 23 cm above ground level and the water droplets released by the spray nozzles reached a height of approximately 150 cm. With each 2 L portion of water or *X. fragariae* suspension pulses of aerosols were generated during approximately 30 s in the first experiment. In the second experiment, due to variation in wind direction, pulses of aerosols of irregular duration were given over a period of 30 to 120 s. Thus, in two days a total of four runs were carried out with *X. fragariae* suspensions being sprayed into the air.

**Fig. 1:**
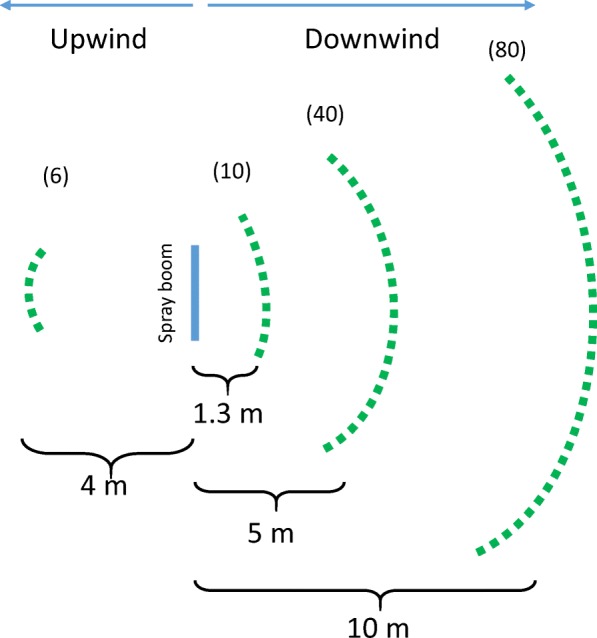
Design of the field experiment. Strawberry plants were placed at one distance upwind and at various distances downwind from the spray boom used to release inoculum (suspension of *Xanthomonas fragariae*). Between brackets: number of plants per distance

Strawberry plants were placed at various distances from the inoculum source in order to establish whether aerolised *X. fragariae* was infectious. Strawberry plants were arranged along three curved lines at various distances downwind and one curved line upwind of a spray boom serving as aerosol source. During spraying tap water with the spray boom (source) six strawberry plants (target plants) were located at 4 m windward and ten strawberry plants at 1.3 m leeward of the spray boom. When *X. fragariae* suspension was sprayed, additionally 40 strawberry plants were placed along curves at 5 m distance leeward from the spray boom and 80 plants at 10 m distance. The target plants were placed along a semi-arc such that each plant was placed at a similar distance from the source. The lay-out of the experiment is described in Fig. 1. The number of plants increased with distance from the source to cover the expansion of the aerosol plume with increasing distance. To mimic recently-fallen rain, the foliage of half of the plants had been wetted with tap water shortly before setting them out in the field. Wet and dry plants were arranged alternating without coming in contact with each other.

At each release of inoculum, a new batch of strawberry target plants was used. The youngest fully expanded leaves, which are most sensitive to infection (Hazel and Civerolo 1980; Hildebrand et al. 2005), had been tagged a few days before the experiments were carried out, to support disease assessments and sampling of exposed leaves later on.

Plants exposed to aerosols were removed from the experimental site before starting the next spray session. They were temporarily placed at a site out of reach of newly generated aerosols. During transfer dripping of water from leaves and contact between plants was avoided. Directly after removal of exposed plants, the same high pressure plant sprayer was used as in the glasshouse experiment to atomize *X. fragariae* suspension on both sides of the labelled leaves of three control strawberry plants until runoff to assess the susceptibility for infection during actual field conditions. At the end of the day all the plants were transported back to the rain shelter and the different groups (distance from the spray boom and leaf treatment) of plants were placed in separate insect-proof cages. Furthermore, a sample of the *X. fragariae* suspension used that day was atomized on the abaxial side of the tagged leaf of three plants kept in a humid chamber to check for the infectivity of the *X. fragariae* suspensions.

### Air sampling and quantification of air particles

Information on the spread of *X. fragariae* in aerosols was quantified by using two methods. Coriolis Micro air samplers (Bertin Technologies, FR) were used to collect aerosolized *X. fragariae* Dylos DC1700 air quality monitors (Dylos Corporation, USA) were used to quantify particles in the air. Equipment was placed at various distances from the inoculum source.

During the spray runs, two Coriolis samplers were situated at 1.3 and 5 m distance leeward from the inoculum source. The capacity of the Coriolis sampler was set at assessing 300 L air per minute. During a period of 3 min starting at the beginning of aerosol generation the microflora present in total of 0.9 m^3^ air at a height of 47 cm above ground level was collected in sterile cones filled with 15 ml RT (quarter-strength Ringer solution with 0.01% Tween20; ThermoFischer Scientific, USA).

The density of the bacterial cells in the air samples collected by the Coriolis Micro air sampler was concentrated 10-fold by centrifugation. Samples were transferred to sterile 50 ml Nunc conical centrifuge tubes (ThermoFisher Scientific), spun at RCF 8867 x g for 10 min at 10 °C in a Fiberlite F-15-6x100y rotor of a SL40R benchtop centrifuge (ThermoFisher Scientific), after which the supernatant was drained and the pellet suspended in 1.5 ml quarter-strength Ringer solution. To check for the presence of *X. fragariae* in the air sample, undiluted and 100× diluted suspension was plated on R2AGRC. After incubation as described above in ‘Xanthomonas fragariae and culturing’ the plates were inspected for presence of Xanthomonas-like colonies. The identity of a random selection of Xanthomonas-like colonies was checked by TaqMan assay. Based on the colony counts the density of *X. fragariae* cfu in air was estimated. In the second experiment at 1.3 m the number of bacterial cfu was higher than 33,000, the upper threshold. For the statistical analysis this upper threshold was used.

### Particle sampling in the air and processing

During the spray runs the extent of decrease in the density of water droplets in the air, due to the expansion of the aerosol plumes and evaporation of water droplets, at 1.3, 5, 10, 25 and 50 m distance leeward from the source, at a height of 45 cm above ground level was recorded with Dylos DC1700 air quality monitors (Dylos Corporation, USA). Furthermore, an air quality monitor at 4 m windward of the aerosol source was used to record the natural background level of particles in the air. The particle counters assess small (> 0.5 μm) particles and large particles (>2.5 μm). Recordings are the average of 10 s measurements. Each minute 6 readings of the number of particles in the air are stored in the data base. During the experiment continuous readings were made. A background number of particles was recorded during the experiment when no suspension was released in the air. When the suspension was released the number of particles recorded peaked. To quantify peaks in the particle counts the number of particles at the time of suspensions release visible as peak values were added (two readings). To compensate for the background particle density naturally present in the air the number of particle counts before the onset of the peak and directly after the release event were subtracted from the peak values. Thus an estimate of the particles in the air due to the release of a *X. fragariae* suspension was calculated.

### Sampling of strawberry target plants and processing

Three weeks after the release of aerolised inoculum and deposition on the strawberry target plants, the tagged leaves and the leaves unfolded just before and just after the tagged leaf, were inspected for the occurrence of symptoms of angular leaf spot. The number of infected plants with and without symptoms was assessed. After the disease assessment the tagged leaves were sampled to test for *X. fragariae* infections. From each target plant that had been located 4 m windward or 1.3 m leeward of the spray boom the complete leaf was cut off and processed. From target plants that had been located 5 or 10 m leeward of the spray boom only one leaflet of the tagged trifoliate leaves was cut off. These leaflets were processed in batches of 4 leaflets for plants at 5 m and 8 leaflets for plants at 10 m.

Each one-leaf or composite leaflet sample was transferred to a universal extraction bag (Bioreba, CH) and crushed using a hammer. Directly after crushing, a volume of Wilbrink’s solution (Koike 1965) equivalent to 5 mL plus 1.3 times the sample weight was mixed through the macerated tissue. Wilbrink’s solution consisted of 10 g L^−1^ sucrose (Sigma-Aldrich), 5 g L^−1^ proteose peptone (Oxoid), 0.5 g L^−1^ K_2_HPO_4_ (Sigma-Aldrich), 0.25 g L^−1^ MgSO_4_.7H_2_O (Sigma-Aldrich), 0.25 g L^−1^ NaNO_3_ (Sigma-Aldrich). To check for the presence of *X. fragariae* in the leaf extract, an undiluted and 100× diluted suspension was plated on R2AGRC. After incubation as described above in ‘Xanthomonas fragariae and culturing’ the plates were inspected for presence of Xanthomonas-like colonies. The identity of a random selection of Xanthomonas-like colonies was checked by a TaqMan assay.

### TaqMan assay

A colony-TaqMan assay was used to confirm the identity of Xanthomonas-like colonies growing on R2AGRC plates seeded with an air sample or leaf extract. Bacterial cells from Xanthomonas-like colonies were sampled with an inoculation needle, suspended in 1 mL sterile water in 1.2 mL collection tubes (QIAGEN). In addition, a so-called bio-TaqMan assay was used to verify the presence or absence of *X. fragariae* colonies on plates on which no Xanthomonas-like colonies were detected during visual inspection. In the bio-TaqMan assay, plates were flooded with 3 mL sterile water and the bacterial colonies dislodged from the agar with the aid of an L-shaped spreader. Depending on the number of colonies 1 mL undiluted suspension, or diluted to a slightly clouded suspension, was transferred to a 1.2 mL collection tube. Next bacterial suspensions were centrifuged for 15 min at 5800 RCF in a 4–15 C centrifuge (Sigma) and 980 μl supernatant was removed from each tube before storage of the pellets at −20 °C until further processing,

DNA extraction from the pellets and the TaqMan assays were conducted as described by Kastelein et al. (2014). Suspensions of which amplification plots showed CT-values >35 were considered negative.

### Data processing and statistics

The data of the disease assessments were used to calculate the disease incidence (expressed as percentage) of symptomatic plants at the three distances leeward from the source of infection. The results of plating leaf extracts were used to estimate the infection incidence (*I*) of strawberry plants using the formula

$$ I=\left\{1\hbox{--} {\left[\left(N\hbox{--} p\right)/N\right]}^{1/n}\right\}\times 100 $$

where *p* is the number of composite samples that tested positive for *X. fragariae*, *N* the total number of composite samples tested, and *n* the number of strawberry leaflets combined into a composite sample (De Boer 2002).

Analysis of variance (ANOVA; Genstat 18.1, VSN International) was used to analyse the effect of *X. fragariae* inoculum density on symptomatic infections of strawberry plants cultivars Elsanta and Sonata under greenhouse conditions. Fisher protected pairwise T-tests were used for evaluating the significance of differences between pairs of averages within cultivars.

ANOVA (with angular-transformed incidences; Genstat 18.1) was used to analyse the effects of distance from the inoculum source and leaf wetness on the occurrence of (symptomatic) infections with *X. fragariae*, Fisher protected pairwise T-tests were used for evaluating the significance of differences between pairs of averages. Water controls were omitted from the analysis.

ANOVA (with log-transformed numbers) was used to analyse the effect of distance from the *X. fragariae* source on the number *X. fragariae* cfu in air assessed with Coriolis air samplers at 1.3 and 5 m. The untreated control as a measure of the background *X. fragariae* population was included in the analysis.

## Results

### Glasshouse experiments

**Fig. 2:**
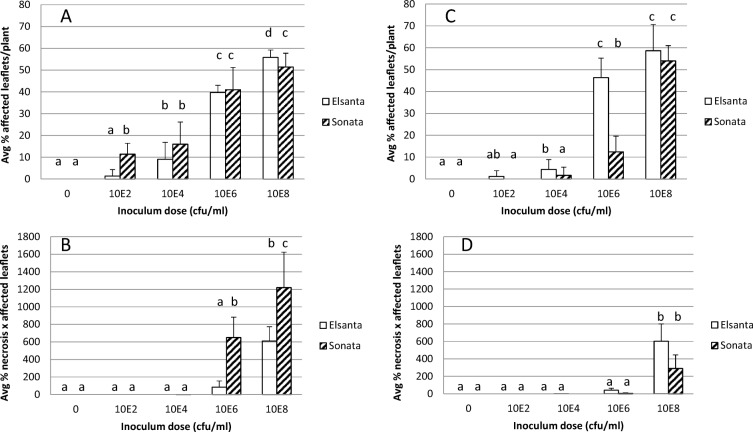
Disease incidences, percentages affected leaflets plant^−1^, determined at 15 dpi and severity indexes, i.e. average percentage necrotic leaf surface times number of affected leaflets plant^−1^, at 40 dpi observed in two-years glasshouse experiments using two cultivars of strawberry plants (cv. Elsanta and cv. Sonata) after spray inoculation with a hundred-fold serial dilution series of a 10^8^ cfu/ml suspension of *Xanthomonas fragariae.* A and B are results obtained in 2013 and C and D in 2014. A and C show disease incidences and B and D severity indexes. Statistical analysis was done per cultivar, per year, separately for incidences and severity indexes (*P* = 0.05). Error bars show standard deviations. Average disease incidence and severity index values for the same cultivar with the same letters are not significantly different (Duncan’s multiple range test, P = 0.05)

In 2013, strawberry plants of varieties Elsanta and Sonata were spray-inoculated with suspensions of *X. fragariae* of either 0, 10^2^, 10^4^, 10^6^ or 10^8^ cfu ml^−1^. At 15 dpi, even the lowest inoculum density resulted in symptomatic plants, although at a low level (Fig. 2a). A positive relation was found between the level of the inoculum density and the percentage of affected plants. At the lowest inoculum density, Sonata had a higher disease incidence, i.e. percentage affected leaves plant^−1^, than Elsanta, but at the highest inoculum density, Elsanta (*P* = 0.05) was more affected.

At the two highest inoculum densities, the disease severity, i.e. amount of leaf necrosis, was higher for Sonata than for Elsanta (P = 0.05) (Fig. 2b). After mock-inoculation, no symptomatic plants were found.

In 2014, the experiment was repeated. In this experiment, only for Elsanta symptomatic plants were found at the lowest inoculum density (Fig. 2-c). In this experiment, overall the disease severity was higher for Elsanta than for Sonata (Fig. 2-d).

### Field experiment

#### Weather conditions during the field experiment

The weather conditions during experimental work in the field are summarized in Table 1. On the first day of the experiment, 25 May 2016, the sky was overcast and no sunshine was observed. On the second day, 13 June 2016, the sky was mostly overcast, but with patches of a thinner layer of clouds, allowing for an insolation that was overall higher than on May 25. On both days the relative humidity during the experiment was around 80% but the days differed in terms of the air temperature during the experiment: around 13 °C on May 25, and 17–19.5 °C on June 13.

**Table 1:** Characteristics of weather conditions during experimental work in the field: temperature (T), relative humidity (RH), wind speed at 2.20 m (U), standard deviation of wind speed (stdU), wind direction (Udir, relative to North) and standard deviation of wind direction (StdUdir)

Date	Time^a^	Spray	T	RH	U	stdU	Udir	stdUdir
			°C	%	m/s	m/s	°	°
25–5-2016	12:35	Water	13.2	80	1.2	0.43	207	6.4
	13:10	*X. fragariae*	13.2	81	2.0	0.29	181	17.5
	13:40	*X. fragariae*	13.1	82	1.7	0.29	219	10.7
13–6-2016	15:20	Water	18.7	80	2.3	0.20	250	15.6
	16:10	*X. fragariae*	19.1	76	4.0	0.69	263	9.7
	17:20	*X. fragariae*	17.3	77	3.7	0.52	251	12.4

Values given are 15-min averages centred around the time of aerosol release, based on data sampled at 1 sample per minute

^a^local time halfway 10–15 min experimental work

Another marked difference between both days was the wind speed at 2.20 m height. During the first day, a low wind speed was measured ranging from 1.2 to 2.0 m/s, whereas on the second day the wind speed, especially during the two *X. fragariae* sprays, was higher at around 4 m/s. The wind direction at 25 May ranged from 180° to 220° during the experiment but was stable at the time scale of an individual dispersion experiment (single release). On 13 June the wind direction was more constant between experiments (250° to 265°) but was much more variable on the time scale of a single release. This experience in the field is not clearly reflected in the standard deviation of wind direction as shown in the table, probably due to the limitations of the wind vane to follow the variations in wind direction. Because of these variable wind directions on the second day, spraying of water and *X. fragariae*-suspension into the air was interrupted several times to achieve a more regular dispersion of aerosols in the main wind direction.

### Splash and aerosol dispersal

The use of a spray boom resulted in the dispersal of both small sized (aerosols) and larger droplets. The larger droplets were dispersed over a distance of at least 1.3 m, as droplets were observed on strawberry plants at that distance. However, after the experiment, no water droplets were found on dry strawberry plants placed at a distance of 5 and 10 m. Obviously, on pre-wetted strawberry plants water droplets were present at all distances.

On both days, no *X. fragariae* was detected by dilution plating in any of the air samples collected with Coriolis samplers prior to release of the inoculum, or when water was sprayed. When *X. fragariae* suspensions were released into the air the pathogen was detected in air samples collected at both 3 and 5 m from the source. The density of *X. fragariae* in air at 1.3 m was 3.0 × 10^4^ cfu L^−1^ and was significantly (F_prob_. = 0.05) higher than at 5 m which was 1.3 × 10 ^4^ cfu L^−1^ (Table 2).

**Table 2:** Densities of *Xanthomonas fragariae* (cfu L^−1^) in air samples collected 4 m upwind (−4 m) and at 1.3 and 5 m downwind from the inoculum, a bacterial suspension released with a spray boom

Treatment	Day 1^a^		Day 2		Days 1 + 2	
−4 m	0	a^b^	0	a^a^	0	a^a^
1.3 m	2.7 10^4^	c	3.3 10^4^	c	3.0 10^4^	c
5 m	9.7 10^3^	b	1.7 10^4^	b	1.3 10^4^	b

^a^The experiment was conducted on two days. The back transformed mean after angular transformation per day (*N* = 2) are shown and the mean of both days

^b^Means without common characters within the same column indicate significant differences between treatments (Fisher protected pairwise T-tests, *P* = 0.05)

On the first day of the experiment, a high linear relation (r^2^ = 0.9958, y = −78,539× + 851,311) was found between the particle counts of the Dylos air quality monitors and the distance from the source in meters (data not shown). On the second day, clear peaks at 1.3, 5 and 10 m were (almost) absent and consequently the particle counts in the peaks could not be assessed reliably. The reason for this absence of a clear peak is two-fold. First, on the second day of the experiment the release was not continuous and spread over a longer time so that the plume of particles was not as clearly defined in space and time as for the first experiment with a continuous release. Secondly, the high variability of the wind speed during the second day (standard deviation of 0.5 m/s as opposed to 0.25 m/s on May 25) may have caused the plume to meander and the particle concentrations to vary at such short time scales that the 10-s mean concentrations did not show a clear peak upon arrival of the plume at the particle counter.

None of the strawberry plants exposed to aerosols during spraying water into the air became infected by *X. fragariae*, nor did plants upwind of *X. fragariae* containing aerosols. However, plants leeward of the source were found infected after release of inoculum, even up to a distance of 10 m from the inoculum source (Table 3).

**Table 3:** Infection incidence of infections of strawberry target plants at 4 m upwind (−4 m) and at 1.3, 5 and 10 m downwind from the inoculum source, a suspension of *Xanthomonas fragariae* released with a spray boom. The strawberry leaves were either dry or wet at the time of aerosol dispersion

Treatment	Distance	*Xanthomonas fragariae*					
		Day 1^a^		Day 2		Days 1 + 2	
dry	−4	0	a^b^	0	a	0	a
dry	1.3	61.0	d	71.0	c	65.8	d
dry	5	8.4	bc	11.9	b	10.1	bc
dry	10	8.4	bc	6.2	ab	7.2	b
wet	−4	0	a	0	a	0	a
wet	1.3	100.0	e	100.0	d	100.0	e
wet	5	16.0	c	21.6	b	18.7	c
wet	10	0.7	ab	9.0	b	3.7	b

^a^The experiment was conducted on two days. The back transformed means after angular transformation per day (N = 2) are shown and the means of both days

^b^Means without common characters within the same column indicate significant differences between treatments (Fisher protected pairwise T-tests)

Generally, the *X. fragariae* infection incidence of strawberry plants was comparable for both days of the experiment and both runs within that day. This allowed us to regard the data of the four runs as data derived from one experiment with four repetitions. The infection incidence of strawberry plants was significantly (F_prob_. <0.05) higher at 1.3 m distance from the source in comparison to infection incidences at 5 and 10 m distance. Furthermore, infection incidences were significantly (F_prob_. <0.05) higher in plants of which the leaves had been wetted shortly before the exposure to aerosols of the pathogen than in plants of which the leaves remained dry, if the distance from the source was not taken into account. The infection incidence of wetted plants was also significantly higher than dry plants at 1.3 m. At 5 and 10 m, the infection incidence of wetted plants was comparable to dry plants (Table 3). Most remarkable is that strawberry plants which had not been wetted became infected by *X. fragariae* at each distance tested. At 1.3 m the dry strawberry leaves were partly wetted by the suspension. However, at 5 and 10 m dry plants were not visibly wetted by the sprayed suspension.

Although leeward from the spray boom many plants got infected, relatively few plants developed symptoms of angular leaf spot. In addition, disease severities were very small (data not shown). The disease incidence varied more than the infection incidence between both days of the experiment (Table 4). On the first day, at 1.3 m the disease incidence was 40% on wetted strawberry plants and 0% on dry plants, whereas in the plants of the second day no symptomatic plants were found on both wetted and dry plants. At 5 m from the inoculum source, the disease incidence on wetted plants was 5.4 and 0% for day 1 and 2, respectively. On dry plants the incidences were 0 and 5.4%.

**Table 4:** Incidence of angular leaf spot of strawberry target plants at 4 m upwind (−4) and 1.3, 5 and 10 m downwind from the inoculum source, a suspension of *Xanthomonas fragariae* released with a spray boom

Treatment	Distance	Symptomatic disease incidence					
		Day 1^a^		Day 2		Days 1 + 2	
dry	−4.0	0	a^b^	0	a	0	a
dry	1.3	0	a	0	a	0	a
dry	5	0	a	5.4	a	1.4	ab
dry	10	0	a	0	a	0	a
wet	−4.0	0	a	0	a	0	a
wet	1.3	39.0	b	0	a	11.0	b
wet	5	1.4	a	5.4	a	3.0	ab
wet	10	0	a	0	a	0	a

The strawberry leaves were either dry or wet at the time of aerosol dispersion

^a^The experiment was conducted on two days. The back transformed means after angular transformation per day (N = 2) are shown and the means of both days

^b^Means without common characters within the same column indicate significant differences between treatments (Fisher protected pairwise T-tests, P = 0.05)

Combining the data of both days of the experiment, symptom expression was restricted to on average 20% of the plants at 1.3 m and 1.4% at 5 m from the source on the pre-wetted strawberry plants (Table 4). On dry plants, symptom expression was found at 5 m distance from the source but not at 1.3 m; the disease incidence at 5 m was on average 2.7%. At 10 m from the inoculum source no symptomatic plants were observed, regardless of the leaf wetness condition of the plant. Symptom expression was more pronounced in the plants of the first day compared to those of the second day, whereas infection incidences bon both days of the experiment were largely comparable.

Seven control plants kept in a humid chamber and spray-inoculated with the bacterial suspensions used for inoculating plants in the field were all infected three weeks after inoculation; two plants were symptomatic. This indicated that the inoculum used was able to cause angular leaf spot. Similarly, in total 12 control plants were inoculated with the bacterial suspension using the spray boom directly after removal of exposed plants. They were all infected three weeks after inoculation and ten plants showed symptoms. This indicated that the conditions in the field were suitable to cause angular leaf spot.

## Discussion

*X. fragariae* released in the form of aerosolized cells can infect strawberry plants, minimally up to a distance of 10 m of the inoculum source. This assessment on risk for infection was supported by the detection of culturable cells of *X. fragariae* in sampled air and by particle counts during the release of inoculum which exponentially decreased with the distance from the source as found on the first day of the experiment. The decrease is a consequence of a Gaussian dispersal of particles in the open air from a point source as described by Spijkerboer et al. (2002).

In the field experiments, the conditions were conducive for infection and symptom development as plants placed at a distance of 1.3 m from the infection source, of which the leaves had been wetted just before the start of the experiment, were found infected. On a relatively high percentage (20%) of these plants symptoms developed. Control plants inoculated in the field with a houseplant mist sprayer developed symptoms of angular leaf spot as well, also indicating that the circumstances in the field were conducive for infection and that the inoculum used was viable.

Control plants placed up-wind from the inoculum source remained free from *X. fragariae*. This indicates that no natural inoculum source was present and no inoculum was disseminated upwind or at least not sufficiently to establish an infection. It further indicates that infections of the target plants were from the released inoculum not from an unknown source in the surrounding. This is supported by the fact that before each release no *X. fragariae* was detected in the air samples collected for 10 min with the Coriolis sampler.

Infections of plants at a short distance of 1.3 m from the infection source may have been caused by aerosols but also by splash dispersal released by the spray boom, as larger droplets were observed on the leaves after the experiment. This may explain the high infection incidence at this distance. At 5 and 10 m distance splash dispersal is unlikely. No water droplets were observed on the dry target plants supporting the lack of splash dispersal.

Experiments were conducted at temperatures of 13 °C during the first and ranging between 17 and 20 °C during the second part of the experiment. According to the literature, the highest number of lesions on leaves are found at moderate temperatures between 16 and 25 °C (Kennedy and King 1962b; Kennedy-Fisher 1997; Hildebrand et al. 2005). Despite the more optimal temperature conditions in the second experiment infection incidences were not higher. Possibly the variable wind during release of the inoculum has resulted in a lower infection pressure. At a low temperature of 5 °C and high temperatures above 30 °C no lesions are formed, but the bacteria will not disappear (Hildebrand et al. 2005; Roberts et al. 1996). During the experiments, a relative high humidity of between 75 and 85% was found. A high humidity is also important for infection, disease development and production of bacterial ooze (Kennedy and King 1962b; Hildebrand et al. 2005).

The infections must have been established within a short time after deposition of aerosols on leaves. There are no indications for an epiphytic phase of *X. fragariae* (Hildebrand et al. 2005; Kastelein et al. 2014), as has been found for a number of other phytopathogenic bacteria, including *Pseudomonas syringae, Xanthomonas axonopodis* pv. *phaseoli* and *Erwinia amylovora* (Hirano and Upper 1983). Here, epiphytes are defined as organisms that can grow or at least reside on the host. In glasshouse experiments in which spray-inoculations of leaves with high densities of *X. fragariae* were conducted, a strong decline of culturable cells in wash water of leaves of at least a 100.000 times was found in the first week after inoculation (Kastelein et al. 2014). Only upon the development of symptoms, population densities in the wash water increased. It is expected that in the field, where *X. fragariae* is subjected to UV radiation and desiccation, population densities will drop even faster than under glasshouse conditions (Beattie and Lindow 1995). Consequently, if air-borne inoculum is deposited on leaves, within a short time free water is required to establish an infection. If free water is present, the bacterium colonizes internal leaf tissues rapidly, as treatment of leaves with a biocide within one hour after inoculation did not result in an effective control (data unpublished). As a consequence, a higher infection incidence for plants was found that were wetted prior to inoculation than for plants kept dry. Nevertheless, infections also occurred on plants kept dry during inoculation. It may be that the aerosols provided sufficient water for the pathogen to migrate through stomata which are identified as main port of entrance for the pathogen (Hildebrand et al. 2005). Alternatively, the relatively high air humidity due to water on irrigation mats and tempered sunshine may have allowed the survival of the pathogen on leaves for a prolonged time. Dew may have provided the water required for the infection. It was found that even at a relative humidity of only 50% a water film can be formed on leaves (Burkhardt and Eiden 1994). Possibly small particles on leaves act as condensation centres during dew formation at a low humidity (Eiden et al. 1994).

Symptom expression depends on bacterial density in plant tissues and growth conditions of the plant. For all plants used on each day of the experiment the growth conditions during the experiment and incubation period were the same. Therefore, we assume that differences in symptom expression within the plants used on the same day were not caused by the conditions, but must be a function of bacterial deposition density. Obviously the infection incidence itself is a prerequisite for symptom expression. Bio-TaqMan analysis revealed that infected plants were present at each distance although the infection incidence became less with distance. Air samples showed that *X. fragariae* was present at 1.3 and 5 m although at a significantly lower density at a lager distance which coincided with a lower infection incidence. In this study, at 10 m from the inoculum source no air was sampled for the presence of *X. fragariae*, however, earlier aerosol experiments showed the presence of *X. fragariae* at that distance (data unpublished). For the first day of the experiment a good relation between the number of small particles in the air and the infection incidence was found. In the second experiment however, no clear peaks were observed in particle sampling, possible due to the higher variation in the wind speed and wind direction during release of inoculum, leading to decrease in infection pressure. Inoculum pressure as expressed by the number of viable cells of *X. fragariae* deposited on the leaves might be correlated to the number of successful infections and subsequently to symptom expression. This was clearly demonstrated in the glasshouse experiments in which the percentage of successful infections was strongly correlated with the inoculum density applied. Furthermore, even at low bacterial counts of 2000 cfu per plant, infection could occur.

This study shows that under field conditions, wind-blown water droplets loaded with *X. fragariae*, can infect efficiently strawberry plants at a distance of at least 10 m from the inoculum source. Under favourable conditions, a low inoculum pressure is sufficient to cause an infection. Management of *X. fragariae* should therefore include roguing of symptomatic plants to reduce the inoculum pressure, avoidance of the release of contaminated aerosols through cultivation practices such as mowing and maintaining sufficient distances between strawberry cultivation plots. The infection risk is less when leaves are air dry compared to wetted plants. Therefore, cultivation practices should be taken preferably in a dry crop rather than in a crop with a wet canopy.
